# No Effect of Insecticide Treated Curtain Deployment on *Aedes* Infestation in a Cluster Randomized Trial in a Setting of Low Dengue Transmission in Guantanamo, Cuba

**DOI:** 10.1371/journal.pone.0119373

**Published:** 2015-03-20

**Authors:** Maria Eugenia Toledo, Veerle Vanlerberghe, Isora Lambert, Domingo Montada, Alberto Baly, Patrick Van der Stuyft

**Affiliations:** 1 Department of Epidemiology, Institute of Tropical Medicine “Pedro Kourí”, Habana, Cuba; 2 Unit of General Epidemiology and Disease Control, Institute of Tropical Medicine, Antwerp, Belgium; 3 Provincial Center of Surveillance and Vector Control, Guantanamo, Cuba; 4 Department of Public Health, Ghent University, Ghent, Belgium; University of Texas Medical Branch, UNITED STATES

## Abstract

**Objective & Methodology:**

The current study evaluated the effectiveness and cost-effectiveness of Insecticide Treated Curtain (ITC) deployment for reducing dengue vector infestation levels in the Cuban context with intensive routine control activities. A cluster randomized controlled trial took place in Guantanamo city, east Cuba. Twelve neighborhoods (about 500 households each) were selected among the ones with the highest *Aedes* infestation levels in the previous two years, and were randomly allocated to the intervention and control arms. Long lasting ITC (PermaNet) were distributed in the intervention clusters in March 2009. Routine control activities were continued in the whole study area. In both study arms, we monitored monthly pre- and post-intervention House Index (HI, number of houses with at least 1 container with *Aedes* immature stages/100 houses inspected), during 12 and 18 months respectively. We evaluated the effect of ITC deployment on HI by fitting a generalized linear regression model with a negative binomial link function to these data.

**Principal Findings:**

At distribution, the ITC coverage (% of households using ≥1 ITC) reached 98.4%, with a median of 3 ITC distributed/household. After 18 months, the coverage remained 97.4%. The local *Aedes* species was susceptible to deltamethrin (mosquito mortality rate of 99.7%) and the residual deltamethrin activity in the ITC was within acceptable levels (mosquito mortality rate of 73.1%) after one year of curtain use. Over the 18 month observation period after ITC distribution, the adjusted HI rate ratio, intervention versus control clusters, was 1.15 (95% CI 0.57 to 2.34). The annualized cost per household of ITC implementation was 3.8 USD, against 16.8 USD for all routine ACP activities.

**Conclusion:**

Deployment of ITC in a setting with already intensive routine *Aedes* control actions does not lead to reductions in *Aedes* infestation levels.

## Introduction

Dengue is, worldwide, the fastest expanding arboviral disease, with an estimated burden of 390 million infections and 96 million clinical cases annually [1]. The agents, four dengue viruses, are primarily transmitted by *Aedes aegypti* and, to a lesser extent, by *Aedes albopictus* mosquitoes. There is no specific antiviral treatment. Immunization has become technically feasible, but in a phase IIb trial the leading vaccine candidate had an effectiveness of between 80 and 90% in preventing DENV3 and DENV4 cases, and just a partial and no effect on reducing DENV1 and DENV2 cases respectively [2]. Even when a vaccine becomes available, attaining adequate coverage in endemic regions will take many years [3]. In the meanwhile, vector control remains the only venue for dengue prevention and in the future it will continue to play a crucial role in complementing immunization efforts.

Currently, for dengue prevention and outbreak mitigation many endemic countries rely on pesticide-based vector control actions that are reactive to the detection of a clinical case or to an epidemic alert. However, the effectiveness of both these strategies is doubtful: the former, applied days after an infection occurs, is directed towards case-houses, which are probably not the major site of transmission [4]; the latter is often launched after the peak of epidemics [5]. On the other hand, pursuing year-round proactive prevention, combining environmental management, larviciding and adulticiding, is costly (up to 24 USD/inhabitant/year) [6] and is difficult to sustain and generally not feasible due to the lack of human and financial resources allocated to the routine *Aedes* control programmes [7].

A few novel, user-friendly vector control tools such as spinosad [8], lethal ovitraps [9] and insecticide treated materials [10–14] have become available over the past decade. Their wider effectiveness and cost-effectiveness is under evaluation. To date, there is evidence that ITC can reduce *Aedes* infestation levels by up to 50%, depending on attained coverage and housing structure [10,12–14]. However, these results were obtained in settings with moderate to high vector infestation.

In Cuba an intensive routine *Aedes* control programme (ACP) has been set up in response to two major dengue epidemics (1977 and 1981) [15]. The ACP covers all of Cuba and implements a combination of vector control actions, which are intensified when the risk of dengue transmission increases. As a result, *Aedes* infestation levels are substantially lower than in other affected Latin-American countries. Nevertheless, outbreaks do occur, be it sporadically [16]. With the aim to prevent these outbreaks, the national control programme is looking for an additional control method that could bring vector densities further down. Insecticide treated curtains (ITC), which showed some promising results in other countries [10, 12–14], were chosen to be explored in a methodologically sound study in order to provide evidence for policy makers.

We report here on a cluster randomized controlled trial that evaluated the incremental effectiveness and cost-effectiveness of ITC deployment in eastern Cuba, a setting with comprehensive routine *Aedes* control and already low *Aedes* infestation levels.

## Materials and Methods

### Study site

The ITC trial was conducted in Guantanamo, a city of 244,000 inhabitants that has an average temperature of 32°C and an average rainfall of 610 mm/year, concentrated in two short wet seasons (April-July and September-October). The average monthly municipal House Index (HI; number of houses with at least one water holding container with *Aedes* immature stages per 100 houses inspected) is low. The HI has oscillated between 0.06 and 0.8% throughout 2003–2007. Still, Guantanamo was affected by dengue outbreaks in 1981 [15], 2001 [17] and 2006 [18]. There has been no evidence of the presence of *Ae*. *albopictus* in the urban area (D Montada, personal communication, 2013).

Although transmission only occurs sporadically, the inhabitants’ knowledge on dengue and preventive measures is high [19] and they are sensitized to the problem. Infestation with *Ae*. *aegypti* is patchy and linked to deficient water supply and suboptimal environmental management. Less than one third of houses (all closed structures, hardly no apartments, very few shops) have permanent water supply and storing water in ground level containers, which are often badly covered and/or in bad condition, is a common practice [19]. The routine ACP, which was intensified during the entire study period in view of a dengue outbreak threat in the eastern part of Cuba, comprises monthly inspection of all premises, temephos application in all useful peri- and intra-domestic water holding containers, elimination of potential household breeding sites with the active participation of the population, complemented with indoor and spatial fogging with cypermethrin every 7–22 days.

### Study design

In January 2009, 12 circumscriptions (the most decentralised geopolitical unit, comprising about 500 houses) were selected in the urban area of Guantanamo amongst the ones with the highest infestation level in the previous two years. circumscriptions with a common boundary with an earlier selected one were not eligible for study inclusion, as some spill-over effect of the purported intervention could be hypothesized [10]. The original study protocol contemplated a latin square design with intensified community participation and ITC deployment as interventions, but during set up community participation was reinforced in the whole municipality of Guantanamo. Therefore, eventually, we randomly allocated the selected circumscriptions to 6 control and 6 intervention clusters, by drawing numbers from a bag. In the control clusters routine ACP activities, as described above, were carried out throughout the study period. In the intervention clusters, the latter were combined with the ITC deployment. The sample size, following Hayes and Bennett [20], permitted to detect a 75% reduction in the HI, with a power of 80% and an alpha error of 0.05, assuming a between-cluster HI variation coefficient of 0.4.

### Intervention

During the month of March 2009, the ACP distributed ITC in all public premises and to households (HH) that had agreed to use them in the 6 intervention clusters. One week before distribution, discussions were held with the inhabitants, which generally perceived that the bedroom and living area were the spaces in the house with most mosquito nuisance and preferred curtains to be hanged there in windows, door openings or on the wall. At that occasion, we also handed out information leaflets and one family member received detailed person-to-person instructions on the use and maintenance of the ITC. The number of curtains distributed (with a maximum of 3) depended on the number of rooms in the house. The ITC were made from PermaNet polyester netting (Vestergaard-Frandsen, Switzerland) treated with a long-lasting formulation of deltamethrin (55mg/m^2^) and coated with a protectant (no details disclosed by the manufacturer) to prevent degradation of the insecticide when exposed to UV light. The manufacturer’s website stated—at the time of the trial—that this material did not require re-treatment and that its insecticidal effect was expected to last for up to 2 years or 6 “standard” washes (http://www.vestergaard-frandsen.com/permanet-curtain-e-brochure.pdf, accessed 22/05/2008). The ITC were purchased from the manufacturer at a unit cost of 2.54 US$ ‘FOB Destination’.

### Data collection and analysis

#### ITC uptake

At distribution, we recorded the number of ITC distributed in each HH of the intervention clusters. Three months after distribution (June 2009), we visited a systematic random sample of 549 HH, observed use of the ITC and explored, with a structured questionnaire, the related perceptions. Continued use of curtains was ascertained during the December 2010 HH visits by routine ACP workers. The number of targeted structures (wall, doors and windows) were not recorded, as these—being structures with very different surfaces—together with the number of ITC distributed would not provide information on the percentage of surface covered.

#### *Aedes* deltamethrin susceptibility

Larvae detected in water holding containers in November 2010 routine ACP visits were reared to adults in the entomological laboratory of the Institute of Tropical Medicine “Pedro Kourí” in Havana. Female mosquitoes, 3 to 5 days old and non-blood fed, were screened for deltamethrin susceptibility using the standard WHO tube bioassay protocol [21,22]. The *Aedes* Rockefeller strain, a susceptible laboratory strain of Caribbean origin, was used as reference. The bioassays (repeated 4 times) were done with 5 replicates of 25 female mosquitoes per bioassay: 4 replicates exposed to insecticide treated paper and one control replicate exposed to non-treated paper. We calculated the proportion of local *Aedes* strain mosquitoes that died at 24h out of the total number exposed. Tests with more than 20% control mortality, if any, were discarded and repeated. When control mortality ranged between 5% and 20%, the mortality was corrected using Abbot’s formula [23].

#### Deltamethrin persistency in the ITC

One year after distribution, we tested deltamethrin persistency and bioavailability in 13 ITC: 2 stored/unpacked new ones and 11 used ones (4 never washed, 7 washed at least once). Tube bioassays were twice carried out at 25 ± 2°C and 75 ± 10% relative humidity, using ten tubes per curtain following the standard WHO procedure [24]. Five to six female mosquitoes were introduced into each tube, which remained vertical throughout the bioassay, and exposed to the ITC sample for 3 minutes. We calculated the average mosquito mortality per curtain and within curtain groups (new, used/unwashed, used/washed). Assays on two untreated curtains served as control and if control mosquito mortality was above 5%, the results were discarded and the assays repeated.

The national pesticide analytical laboratory (Instituto de investigaciones de Sanidad vegetal, Havana) determined the deltamethrin concentration in five ITC using High Performance Liquid Chromatography (HPLC), following standardized procedures (http://www.cipac.org/document/prepublished_methods/CIPAC_333_extension_DM%20in%20PE%20netting_090526.pdf). Five ITC were purposively selected—one new and 4 used ones having *Aedes* mortality results in the tube assay of > 95% (1), between 60–70% (2) and < 10% (1)-, and tested. We calculated the Pearson correlation coefficient between bioassay *Aedes* mortality rates and HPLC results of the selected 5 ITC.

#### Entomological impact

The routine ACP conducts once a month entomological surveys in all dwellings of the municipality. Its records combine the observations of the vector control workers, who inspect 100% of the houses, and of quality control inspectors, who re-visit a systematic sample of 33% of the houses. In the month of distribution, and the month before and after distribution, quality control inspectors re-visited 100% of the houses in the intervention and control clusters. For all immature stages found, species was identified. We extracted from the routine ACP records, for Guantanamo municipality as a whole and for each study cluster, information on the number of houses inspected monthly, the number of positive containers and the number of houses with at least one container positive for immature *Aedes* stages in the period April 2008—September 2010 (i.e. 12 months before and 18 months after ITC distribution).

*Aedes* infestation levels were the primary trial outcome. We calculated House Indices (HI) and Breteau Indices (BI) per study cluster per month. We analysed the intervention effect over the 12 and 18 month period starting from April 2009, the month of the first house inspection cycle after ITC distribution. For descriptive purposes, we graphed the average monthly HI per trimester (with 95% CI) for each study trial arm and for the municipality of Guantanamo. To evaluate the effect of the intervention, we constructed generalized linear random effect regression models with a negative binomial link function. We evaluated the period effect (pre- and post-intervention) and the trial arm effect (intervention or control clusters) on the monthly study cluster level HI and BI. This model takes into account the nature of the data (repeated measurements in each cluster) and allows for the assessment of a possible interaction between period and trial arm effect, which captures the effect of the intervention.

#### Cost evaluation

We collected the costs of the routine ACP activities and of ITC implementation in 2009, taking the point of view of the routine ACP. Data on resource utilization and expenditures were extracted from bookkeeping records and activity registers of the routine ACP and from the expenditure accounts of the ITC deployment study. Expenditure and costs were classified in personnel, materials and supplies, and capital cost items, according to Johns *et al*. [25]. We distinguished opportunity expenditure (labour, consumables and capital cost routinely incurred by ACP, but now devoted to ITC implementation) from incremental expenditure.

Since, according the manufacturer, curtains are expected to last for two years, we treated all expenditure for ITC implementation as capital and annualized at 3% interest rate. Costs were calculated at 2009 prices and converted from the national currency to US$ following Jose Luis Rodriguez (http://www.cubadebate.cu/opinion/2013/12/02/hoy-es-posible-comenzar-a-revertir-la-dualidad-monetaria-en-cuba/), using an exchange rate of 1 CUP = 1 US$ for goods and 10 CUP = 1 US$ for salaries.

We used Microsoft Excel 2010, Stata IC10 and SPSS 21 for the analysis.

### Ethical considerations

The study received clearance from the ethical committee of the Institute of Tropical Medicine “Pedro Kouri”, from the national health authorities and from the Institutional Review Board of the Institute of Tropical Medicine, Antwerp (nr 08254622). Community representatives from each participating circumscription approved the intervention and written informed consent was obtained from every HH included in the study. The ITC were made from material that has been approved for use as bed nets by the World Health Organization Pesticide Evaluation Scheme (WHOPES). No patients were included in the trial. We misinterpreted the length of the transition-period for retrospective registration, and the trial was eventually registered at the Current Controlled Trials register (number ISRCTN37433764) in March 2011, one year before the expected end date of study. The authors confirm that all ongoing and related trials for this intervention are registered.

## Results

**Fig 1:**
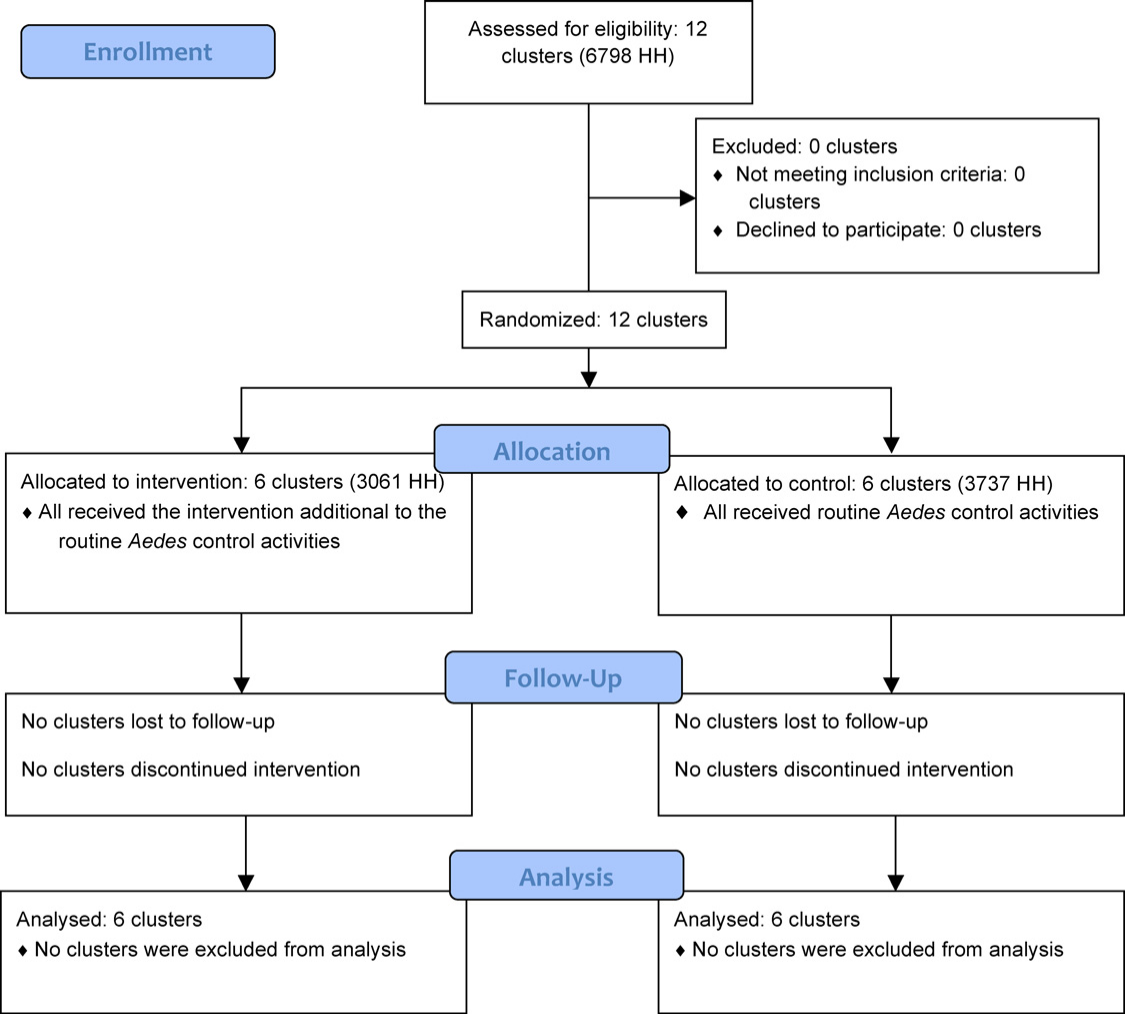
CONSORT flowchart.

A total of 6798 HH were included in the study: 3061 HH in the intervention clusters and 3737 HH in the control clusters. All clusters received the intervention as randomized and were followed up as planned (Fig. 1).

In March 2009, a median of 3 ITC per house (P25 2; P75 3) were distributed in 3015 of the 3061 HH in the intervention clusters (98.4%). Three months after distribution, the coverage—evaluated in a sample of 549 HH—remained very high: 99.1% of the HH (95% CI 97.9–99.6%) still used at least one ITC. In 12 (2.2%) of the HH one or more of the received ITC had been removed, reportedly because ITC were damaged or given to a friend or family-member. Only 4 persons reported a side effect: minor allergic reaction (temporary [less than 48 hours] itching of handpalms) after handling the newly distributed ITC. In 35.5%, 62.7% and 36.4% of the HH, one or more ITC were hanging in an inside door, on the wall behind the bed in the main bedroom and in a window, respectively. In 97.1% of HH, the ITC had no or little direct exposure to sunlight. ITC were hanging open during the day of survey in 96.3% of premises. Users’ perceptions of benefits and disadvantages are summarized in Table 1. In December 2010, use of at least one ITC was observed in 97.4% of HH in the intervention clusters.

**Table 1:** Perceptions on Insecticide Treated Curtain deployment in 544 households, out of 549 sampled, that are still using them 3 months after distribution, Guantanamo, June 2009.

		N (%) (95%CI)
Benefits *	Less mosquitoes entering the house	339 (62.3) (58.2–66.4)
	Fewer mosquitoes in the house and yard	152 (27.9) (24.2–31.7)
	Curtains look nice	53 (9.7) (7.2–12.2)
Disadvantages	Difficult to keep the ITC hanging open during the day	0 (0)
	Curtains are easily damaged	23 (4.2) (2.5–5.9)
	Curtains become easily dirty	43 (7.8) (5.6–10.2)
Probing whether mosquito nuisance declined	Effect perceived on nuisance	337(61.9) (57.9–66.0)
	No effect perceived on nuisance	205(37.7) (33.6–41.7)
	No opinion	2(0.4) (0.0–0.9)

* *first spontaneous answer*

A 99.7% (399/400 exposed mosquitoes) mortality rate in the tube bioassays indicated that *Aedes* mosquitoes strains in Guantanamo were susceptible to deltamethrin. The persistency of deltamethrin in the ITC was: 98.0% (95% CI 94.9–99.2%) *Aedes* mortality in the bioassay on never used curtains; and on ITC used for 1 year 73.1% (95% CI 63.1–83.1%) and 59.1% (95% CI 28.3–89.9%) on never-washed and washed, respectively. No assay results had to be discarded, as the control mosquito mortality stayed below 5%. HPLC analysis on the purposively selected ITC with low and high persistency of deltamethrin according to the bioassay tests, revealed deltamethrin concentrations ranging between 4 and 68 mg/m^2^, which were congruent with the bioassay results (Pearson correlation coefficient 0.69).

**Fig 2:**
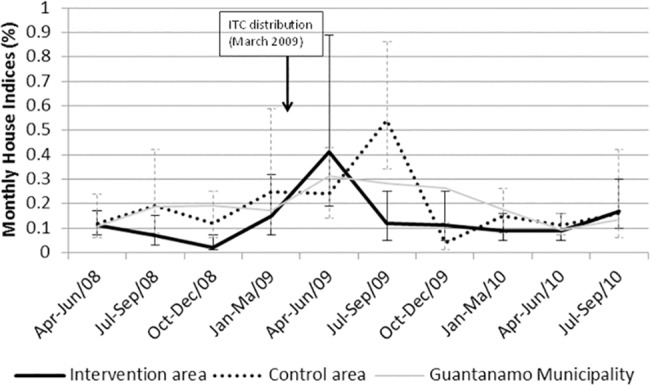
Monthly House Indices, averaged by quarter, in intervention and control clusters and in Guantanamo municipality (April 2008-September 2010). Intervention area (black bold line); Control area (black dotted line); Guantanamo municipality (grey line).

The House Indices in Guantanamo municipality and in the intervention and control areas varied considerably over time (Fig. 2). Only in the trimester July-September 2009 (4–6 months after ITC distribution) was there a difference between the intervention and control arms (HI rate ratio of 0.31 (95% CI 0.11–0.87)). Taken over the entire post-intervention period, there was no significant effect of the ITC on *Aedes* infestation levels (Table 2): corrected for pre-intervention infestation, the HI rate ratio—clusters with against without ITC—was 1.07 (95% CI 0.42–2.37) and 1.15 (95% CI 0.57–2.34) over the 12 and 18 months post ITC distribution respectively (the BI rate ratio results are not shown, but are very similar to the HI results). In absolute terms, the difference of average differences in pre- and post-intervention HI between control and intervention clusters was—0.01 (95% CI-0.07–0.04).

**Table 2:** The effect of Insecticide Treated Curtain deployment on House Indices^§^ (%), Guantanamo, 2008–2010.

Period	Mean HI (95% CI)		Rate ratio Intervention:control (95% CI)*	Adjusted Rate Ratio Intervention:Control (95%CI)**
	*Intervention clusters*	*Control clusters*		
Pre-intervention (April ‘08-March ‘09)	0.09 (0.05–0.15)	0.17 (0.09–0.32)	0.86 (0.42–1.79)	-
12 month post-distribution (April ‘09-March ‘10)	0.18 (0.10–0.32)	0.25 (0.17–0.37)	0.85 (0.46–1.59)	1.07 (0.42–2.37)
18 month post-distribution (April ‘09-September ‘10)	0.17 (0.10–0.27)	0.21 (0.14–0.30)	0.92 (0.56–1.52)	1.15 (0.57–2.34)

* *estimated with a generalized linear random effect regression model with negative binomial link function*

** *adjusted for pre-intervention infestation*

^§^ *House Index = number of houses with at least 1 container with Aedes immature stages/100 houses inspected*

The annualized cost per household of ITC implementation was 3.8 US$ (Table 3), of which 84.0% were incremental costs. Curtain purchase cost accounted for 74.3% of the total cost. The yearly cost of the routine *Aedes* control activities in Guantanamo was 16.8 US$ per household. Of this latter amount, 60.8% was for larval stage and 39.2% for adult stage *Aedes* control.

**Table 3:** Opportunity expenditure, incremental expenditure and annual average cost per household (US$) of Insecticide Treated Curtain implementation in the intervention clusters, Guantanamo, 2009.

Cost items	Opportunity expenditure	Incremental expenditure	Total expenditure (%)	Annualized cost per household
Labor	2,309.4	0.0	2,309.4 (10.0)	0.38
ITC	0.0	17,041.5	17,041.5 (74.3)	2.83
Other materials	1,135.0	2,202.0	3,337.0 (14.6)	0.55
Capital	250.0	0.0	250.0 (1.1)	0.04
**Total**	**3,523.4**	**19,243.5**	**22,937.9 (100.0)**	**3.80**

## Discussion

There was no effect on dengue vector infestation levels of ITC deployment on top of routine vector control activities in Guantanamo, Cuba, despite susceptibility of the local *Aedes* strain to deltamethrin, persistence of the insecticide in the ITC and very high sustained ITC utilization.

Main strengths of our study are the cluster randomized design, which prevents possible confounding by ecological, climatic and other (unknown) factors, and a monthly follow-up of effects until one and a half year post-distribution. Dengue is not endemic in Cuba, but occurs in small, localized outbreaks years apart [16] and we could not evaluate the effectiveness of ITC deployment on disease transmission. Instead, we used *Aedes* immature stage infestation levels, also because monitoring adult *Aedes* populations has low reproducibility [26]. It is, however, a relative weakness that our study relies on entomological data collected through the routine surveillance system. Apart from limited overall non-differential underreporting [27], we cannot exclude that the vector control workers—keen to appraise the effect—may have somehow intensified the search for breeding sites in the intervention and control clusters. On the other hand, we obtained, monthly repeated measurements, which are seldom collected but permit adjusting for small scale temporal variability in the analysis.

The insecticide persistence in the ITC is in line with earlier reports [28]. However, curtain uptake and use were high and sustained, in contrast to more modest coverages and rapid declines in previous effectiveness trials in Venezuela and Thailand [13,14]. We hypothesized that engaging the community increases the acceptance of a control tool [29] and enhances sustainability [30]. One week before ITC distribution, all households received a visit to discuss the intervention and to negotiate where and how to hang the curtains. Each HH also received an information leaflet where the use, advantages and side effects of ITC were made explicit. At the same time, sustained high coverage might also be explained by the perception in more than half of the users that curtains reduce the mosquito nuisance, by the awareness of an increasing threat of local dengue transmission and by the monthly routine house inspections of the vector control workers.

Our main findings also contrast with these studies that demonstrated 50% and 68% reductions of entomological indices when 50% and 70% of HH use an ITC, respectively [13,14]. However, the context of the present trial is different. Firstly, the vector infestation levels are lower in Guantanamo than in the areas where the tools were previously tested [10,12–14] and than in most dengue endemic regions. For that matter, Guantanamo represent the most challenging scenario for layered intervention impact.

Three ITC per HH, a likewise average coverage as in other ITC-trials [14, 31], also might just not be sufficient to impact on vector densities because of a too low probability of mosquito insecticide contact. Secondly, the effect of introducing an additional control tool will depend on the interventions already in place. In Guantanamo, such “other” actions were already intensively combined in the routine control programme and we evaluated the additional effect of ITC implemented on top of the existing activities. The effectiveness of concomitant interventions directed at adult vector stages and implemented in a prospective and controlled way has not been studied yet in dengue control and hardly in vector borne diseases in general. For malaria however, no significant benefit was observed when adding indoor residual spraying to insecticide treated material (bed net) deployment, two malaria control methods that are effective when used in separation [32, 33].

Little or no effect of ITC on dengue vectors has been observed in 3 other studies, conducted in other settings with fewer and far less intensive routine activities, with other hypotheses offered as explanation. In Southern Thailand, an open housing structure was seen as the main reason [12]. In Venezuela and Mexico [10], a ‘spill-over’ effect from the ITC intervention clusters to the adjacent control clusters was presumed. In Guatemala, additional actions targeting the main breeding sites in the intervention arm only, confounded the results 31]. Neither of these can explain our findings, as the housing structure in Guantanamo is closed, the intervention and control clusters were separated by at least one house block and the routine ACP applied the same control measures, with universal coverage, in the intervention and control clusters.

The non-annualized cost of 7.6 US$ per household covered of ITC implementation in our study (3.8 US$ when annualized) is comparable to 6.9 US$ estimated in a similar study in Venezuela [34], but the prices of the cost items were somewhat different. A study in Guatemala [31] estimated the costs of distributing ITC and a few insecticide treated drum covers, combined with treating the most productive container types with temephos, at 5.3 US$ per HH. The cost of ITC itself was not included in this estimate, nor reported. However, curtains are expensive and made up, in Guantanamo, 74.3% (5.6 US$) of the total cost per HH of the intervention. At any rate, the incremental expenditure is high, even in relative terms for an already intensive control programme. Also, in settings, where the investment per household by the routine vector control programme is still rather modest, as in most dengue endemic countries, ITC implementation is not a plausible, financially attractive option from programme managers perspective and probably also not an efficient one [34]. In view of the lack of marginal effectiveness, ITC deployment on top of an intensive vector control programme is obviously not cost-effective.

Studies like the present one undoubtedly support selecting cost-effective strategies in dengue control programmes and facilitate policy making at the local level. However, the question remains how to further reduce *Aedes* infestation and dengue transmission at global level. Future research will have to address the effectiveness of new tools for adulticiding, including ITC, in an array of contrasting environments. Most importantly empirical studies should sort out where—context—and when—timing—and how—targeted at high risk areas or with a blanket approach—existing and new dengue control tools can be most efficiently combined.

## Supporting Information

S1 CONSORT Checklist (DOC)

S1 Protocol (DOC)
