# Supplementary material for: No Effect of Insecticide Treated Curtain Deployment on Aedes Infestation in a Cluster Randomized Trial in a Setting of Low Dengue Transmission in Guantanamo, Cuba
Source: PLoS One. 2015 Mar 20;10(3):e0119373. doi: 10.1371/journal.pone.0119373 (PMC4368727; doi:10.1371/journal.pone.0119373)
Supplement: S1 Protocol — (DOC) [file pone.0119373.s002.doc]

# PROTOCOL

# I. Generalities

# **A. Title project** : Acceptance and Cost-effectiveness of insecticide treated curtains in areas with low *Aedes* Infestation levels

## B. Collaborating institutes and investigators:

Principal Investigator: Patrick Van der Stuyft, ITG

Collaborators :

- ITG: Veerle Vanlerberghe
- IPK: Maria Eugenia Toledo, Alberto Baly
- National Vector Control programme: Juan R. Vazquez Canga, Ms. Alina
- Programa provincial de Control de Vectores, Guantánamo: Juan Ramon Benitez, Damary Gomez, Maritza Rodriguez

# II. Introduction and problem statement

Dengue is a viral vector-borne disease, predominantly affecting the urban areas but extending into more populated rural areas. The main insect vector is the highly synanthropic *Aedes aegypti* mosquito. The vector breeds in water storage containers and other deposits containing relative clean water, and bites and rests in and around human dwellings. Attempts at eradication failed during the 1960s in Latin America so today more than 50% of houses are infested with *Aedes* larvae in many endemic areas (1;2).

Dengue prevention has long depended on control of immature vector populations either by insecticidal treatment (larviciding) or removal of breeding sites and occasionally, biological control. These interventions showed a variable success and are in most of the countries difficult to sustain. Control of immature stages impacts only on vector densities and not on mosquito longevity or other key parameters affecting dengue transmission: targeting adult vectors is more likely to reduce both transmission and mosquito density. Insecticide-treated materials (ITM), highly effective in preventing malaria and other nocturnally transmitted vector-borne diseases, have shown a first proof of efficacy on dengue vector densities and potentially on dengue transmission in Venezuela and Mexico (3). These ITM are generally well accepted by communities and additionally: a) insecticide-treated curtains (ITCs) might be hung inside houses (in doorways or as screens) avoiding harmful UV light; b) other insecticides and novel biological insecticides may be safely applied to netting, thus providing a means of managing pyrethroid resistance; c) interest from new and existing industry partners will result in new products to meet future control needs. The WHO Dengue Scientific Working Group of 2006 identified the development and evaluation of ITM as a primary global research stream.

New vector control tools should be user-friendly and not require much additional work from householders; they should be affordable, safe and effective in reducing vector densities below threshold levels estimated by the "pupae per person index"(4;5). There are many examples of potentially effective disease control products that have had only limited impact on the burden of disease because of inadequate implementation resulting in poor access. Implementation research has to help ensure that proven control products have the intended health impact. Implementation or distribution of tools of public health interventions consists of different components: geographical availability of product, economical affordability, acceptability of intervention and tool, demand for intervention, distribution strategies, …

Many diurnal human activities take place outside the house (schools, factories, …) and many individuals are potentially exposed to dengue transmission away from the household, therefore these places will be included in the intervention where possible. The difficulties and opportunities of inclusion of these sites in the tested strategies will be evaluated.

***Directly addressing these aspects, we will undertake a study, investigating the interaction between an ITM distribution (adult targeting) and community-based environmental management strategy (immature stages targeting) in a municipality of Cuba, where the baseline infestation already reached low levels, but where transmission still occurs. In this project, the tools will be delivered for free, so we will focus our research on the uptake, acceptability, effectiveness and cost-effectiveness of tools with or without a combined community-based environmental strategy.***

# III. Research objectives and hypothesis

## III.A. General Objective

Evaluate the acceptance, effectiveness and cost-effectiveness of Insecticide treated curtains (ITC), as a single *Aedes* control strategy or as a combined strategy together with community-based environmental management.

## III.B. Specific objectives

1. Evaluate the acceptability and uptake of tools in the combined (ITC and community-based environmental management) and single strategies
2. Evaluate the effectiveness of ITC in the control of *Aedes aegypti* and its added effect when applied in combination with other strategies
3. Evaluate the cost-effectiveness of an ITC intervention as a single and combined strategy

# IV. Methodology

## IV.A. Description of study and Research design

A. Description of study and study areas
Area Sur and Centro of Guantanamo were identified as the areas where the study sites will be selected from. Both areas have high infestation-levels in comparison to other areas of Guantanamo municipality.

The circumscriptions (lowest geopolitical unit, containing approx. 500 houses) were identified as unit of intervention and of analysis of effectiveness.

Circumscriptions with the highest infestation –levels will be selected and afterwards the allocation to control or intervention group will be on a random basis. The circumscriptions with a common boundary will be eliminated from the study, as a certain spill-over effect is expected (3).

Design:

Cluster-randomized controlled trial, Latin square design

|  | ITC Distribution | No ITC distribution |
| --- | --- | --- |
| Assisted CB environmental management | Group Intervention 2 | Group Control 2 |
| No assisted CB environmental management | Group Intervention 1 | Group Control 1 |

*Group Intervention 1*: Distribution of Curtains, no assisted community-based (CB) environmental management

*Group Intervention 2*: Distribution of Curtains + assisted CB environmental management

*Group Control 1*: No curtains, no assisted CB environmental management

*Group Control 2*: No curtains, assisted CB environmental management

Sample size estimation (based on the effectiveness of curtains measured through entomological indices):

The cumulative house index over 1 year is estimated at 2%.

We estimate that we can decrease the infestation-levels with 75% in the ITC intervention strategy in comparison with no intervention. Based on the Hayes and Bennett sample size estimation (6), we need 6 clusters of 500 houses in each arm (‘ITC distribution’ and ‘no ITC distribution’) to demonstrate the effect of the curtain-intervention (power of 80%). The subdivision of each arm in 2 groups makes it possible to 1) measure the influence of adding an ITC strategy on top of the community involvement process and 2) the influence of community involvement on the ITC uptake.

(Annex 1)

Duration of study: Primary and secondary outcomes will be evaluated over a period of 2 years.

Outcomes:

*Primary outcome:*

Effectiveness: cumulative house index (on year basis) and other *Aedes* infestation indices

*Secondary outcomes:*

- Uptake and use of IT curtains

- Satisfaction with IT curtains

- Level of community involvement reached

- Change in intra- and extra domiciliary risk for *Aedes* infestation

- Cost-effectiveness of IT curtains

## **B. Design of intervention:**

Curtains:

The insecticide-treated curtains (ITC) to be used are made from long-lasting, insecticide treated (pyrethroid deltamethrin is applied during manufacture) polyester netting that requires no re-impregnation (PermaNet®; Vestergaard-Frandsen company). PermaNet materials are special UV protected and retain their insecticidal properties and efficacy for about 2 years (information from producer). The material has been approved by WHOPES for use as bednets. Three insecticide impregnated curtains (ITC) will be distributed in each house: 2 window curtains, cut to fit the window opening (by preference not on the window towards the street, due to street-dust and hence frequent washing needed) and 1 door curtain (to be placed in a door-opening inside the house or on the closet or on the wall where no or minimal sunshine will touch the material - if a curtain is already hanging, the project-curtain can be attached to it).

All curtains will be white patterned netting and have a 1.6m width*2.2m height size (so that people can adapt them according their window and door size).

Before distribution the consent of the users will be procured in an informed consent-form designed for this project (see annex 2).

The curtains will be distributed with an accompanying promotion campaign: a Frequent Asked Questions document (FAQ) is already elaborated (see annex 3). It will be adapted and used during this campaign, together with other context-specific promotion materials.

The curtains will be distributed to the houses, but also to public places, as working areas, schools, cultural centra will be included as far as possible.

A replacement of curtains will be foreseen for the curtains in very bad condition. If new persons are coming to live in the circumscription during the study, they will be approached for their interest to be included in the study.

Assisted community-based environmental management

Main components of this strategy:

(a) establishment of a formal task force at circumscription level to drive the environmental management activities. This group identifies, designs and implements local environmental management actions, by actively involving the community

(b) intersectoral coordination between this task force and the existing governmental structures and sectors (education, promotion, water-supply, municipal services, …)

(c) close collaboration between these task forces and the health system (especially the vector control programme).

Besides of the routine vector control programme, the assistance to set-up this intervention will be done by a local research group who will be responsible for its implementation, the coaching of the local task forces, coordination with the local health authorities, documentation of process and organization of training sessions according to the needs of the actors involved.

Distribution models for distribution of ITC

*Group I intervention*: the ITC will be distributed by the ‘operarios’ of the municipal routine vector control programme during their routine inspection visits. The communication campaign that will accompany the distribution will be designed by the municipal/provincial dengue control programme.

*Group II intervention*: The curtains will be distributed by the ‘trabajadores comunitarios’ and their local existing networks. The aim is that this model of distribution is a participatory one, where the way of distribution, promotion and communication is discussed within the existing community networks.

The Control-sites

In all areas, the routine control activities will be continued (only strategy in Group Control 1). The standard control activities are carried out by the programme’s vector control workers: entomological surveillance and source reduction through periodic inspection of houses, larviciding (with temephos) of water-holding containers, selective adulticiding (cipermethrine and clorpiriphus) when *Aedes aegypti* foci are detected, providing health education, promotion of community based environmental management and enforcing mosquito control legislation through the use of fines.

C. Study components, data collection and analysis:

1. Baseline investigation:
   1. Entomological survey:

Existing of larval and pupal surveys; the larval surveys will be conducted as in the routine system (exhaustive inspection of all houses and containers on the presence of immature stages of Aedes). The traditional Stegomyia indices will be calculated: the House Index (HI) and Breteau index (BI). The pupal surveys will consist of direct inspection inside and around all the houses following the recommendations of Focks (5). Each survey will consist of a brief questionnaire and the inspection of domestic/peridomestic areas of each house looking for potentially water-holding containers and the complete collection of mosquito pupae. The total positive containers for Ae. aegypti and their relative contribution to total pupal production will be recorded and computed. The number of persons per house will be also enquired during the application of the questionnaire for the construction of Pupal Index per inhabitant (PPI) at the cluster level and averaged on group level.

The data (positive containers) will be retrieved from the routine entomological surveillance (in the regular cycles done by VC workers), added with the foci encountered in a control visit (by independent external control workers) 1 day after the routine visit.

- 1. Opinions and expectations on ITC in the clusters that will receive ITC: Focus Group Discussions with groups of men, women and leaders separately (3 FGD per intervention group, 12 in total). Subjects: opinion on existing dengue control measures and preferences, expectations concerning the ITC, willingness to use the ITC, appropriateness of distribution channels of vector control tools. (Annex 4)
  2. Design of distribution and communication strategies: through expert- interviews:
     1. Communication experts: what are the messages that are best promoting dengue control strategies? Which are the best communication channels? How to organise best promotion in a continuous way? How to organize communication and promotion messages in a participatory way?
     2. Provincial/municipal vector control programme and community networks: exploration of best adapted and efficient distribution strategies (annex 5)
  3. Evaluation of community involvement level in dengue control activities at baseline in the 4 groups.
     1. Through Focus group Discussions with community networks and inhabitants, and through in depth interviews with inhabitants purposively sampled, data will be collected using the Rifkin-tool (already adapted to local context by Dr. D. Gonzalez, see annex 6 & 7) (intervention and control groups).
     2. Revision of documents:
        - Coverage of community workers
        - Existence of Community network
        - Revision of needs identification and plan of action (for environmental management activities) done by Community network
        - Revision of report of activities and risk monitoring form of Community network

1. Monitoring:
   1. Entomology:
      1. The same measurements as in the baseline entomological study will be done once intervention started (House Index (HI), Breteau index (BI), Pupal Index per inhabitant (PPI)). The frequency of the surveys will be according the cycles taking place by the routine control programme, with at least one survey per month.
      2. Repeated blocks
      3. GPS geo-referenced positioning system for follow-up of foci
   2. Epidemiology of clinical Dengue cases if an epidemic presents itself in the period of study
   3. Observation of main intra and extra domiciliary risks at household level (data collection through an existing monitoring system)
   4. Uptake and use of curtain

Uptake and its seasonal variances will be evaluated every 4 months (3 X/year) over a period of 2 years. During observation-visits in all houses, these aspects will be evaluated (Observation guide: annex 8).

- 1. Registration of executed vector control activities: information obtained from community perspective (Questionnaire: annex 9)

Every 4 months, one in 30 households will be questioned concerning the vector control activities that were realized in the previous period.

.

1. Mid-term assessments
   1. Evaluation of acceptance, use and satisfaction (curtains and implementing strategy) through household surveys, 3 and 18 months after distribution (in a sample of 330 houses/intervention arm (110 houses/circumscription, systematic random sampling)) (data collection form: annex 10)
   2. Evaluation of the adoption of the model of implementation/communication: (process evaluation) through key informant interviews (18 months after distribution)
   3. Evaluation of level of involvement of community in control actions (18 months after distribution)
   4. Persistency testing of the curtains (monitoring insecticide content of PermaNet curtains over time): This will be tested by the standard WHO cone bioassays on a sub-sample of 10 curtains after 6 months of use and subsequently at approximately 6-month intervals. (Will probably be done in LSHTM, London or LSTM, Liverpool)

**Analysis**: Two major subjects:

1. The influence of (1) individual and household characteristics, (2) model of distribution and communication and (3) community involvement level will be evaluated on the effectiveness and ITC uptake measures.
2. The influence of adding an ITC strategy will be evaluated on the community involvement process and level reached.
3. Cost-effectiveness evaluation

We collected already the financial cost of the vertical programme during the two last years preceding the intervention and will calculate the average cost per house inspected and treated.

For each arm, we will multiply the number of houses inspected and treated annually during the study by the corresponding average cost described above, to calculate the total annual cost of the routine programme per arm.

We will calculate the total economic cost of the new control tool (curtain): The financial cost of the routine programme and economic cost of the community participating in the distribution, installation and maintenance of the tools; and the economic cost of the community participating in collective vector control activities.

The total annual cost of the different arms can be obtained adding the cost of the routine programme and the economic cost of the implemented strategy.

We also will calculate the average and marginal cost per house within arms and incremental cost per house between arms.

The activities of vertical programme will be costed prospectively during two years of the study, as well as the cost regarding the distribution of the tools itself. The cost information about the installation, maintenance, and participation in collective activities will be followed by community vector control workers during their routine activities and the corresponding questions will be included in the household surveys of uptake (taking place at least every 4 months) or acceptability, in 1 out of the 30 houses in all clusters.

We will obtain the effectiveness measure from the entomological study and use the difference between the baseline data and the data for the subsequent periods as effectiveness measure.

Average cost-effectiveness will be calculated, dividing the cost by the effectiveness estimate for the corresponding period; also, incremental cost-effectiveness will be calculated by dividing the difference in total cost by the difference in effectiveness between the strategies.

The robustness of the cost effectiveness estimate we will evaluate performing the sensitivity analysis, varying the price of the community time, and using the variation observed in the correlation between the coverage of tools and the corresponding entomological outcome.

## IV.D. Quality Control

1. Data collection in the routine entomological surveillance system of Vector Control programme:
   1. Revision of coverage of programme workers prior to and during project
   2. Revision of quality of routine work prior to and during project
   3. Control visit of an extra entomological team in all houses 1 day after the routine inspection done by the campaign workers
2. Data entry: a double data-entry will be performed in Access, questionnaires already coded
3. The evaluation of the Community involvement, obtained through Focus Group Discussions with the community networks, will be counterchecked with documental revision of their work and achievements.
4. The information about the vector control activities in houses given by households at individual level will be compared with the information collected during direct observation of the environmental risk situation of the houses.

## IV. E. Ethical concerns

The protocol will be revised by the scientific board of the Institute of Tropical Medicine, Antwerp, Belgium, the ethical committee of the University of Antwerp, Belgium and the ethical committee of the Institute of Tropical Institute (IPK), Havana and the Cuban health authorities.

Informed consent from local authorities and community representatives for distributing new vector control tools in the area will be obtained. Informed consent of interviewees and Focus group discussion participants will be taken. The researcher will explain the research project: the objectives, the funding, methods, the involvement of the participants and the length of time of involvement, and plan on the use of the research outcomes, including how these will be disseminated. All this information will be placed in the Informed Consent form (annex 2) that will be written in Spanish.

The confidentiality of data will be ensured during data management and analysis. Participants will not be identified in any written or oral report, especially in relation to the data they provided.

## IV.F.Time plan

| Activities | 1 | 2 | 3 | 4 | 5 | 6 | 7 | 8 | 9 | 10 | 11 | 12 | 13 | 14 | 15 | 16 | 17 | 18 | 19 | 20 | 21 | 22 | 23 | 24 | 25 | 26 | 27 | 28 |
| --- | --- | --- | --- | --- | --- | --- | --- | --- | --- | --- | --- | --- | --- | --- | --- | --- | --- | --- | --- | --- | --- | --- | --- | --- | --- | --- | --- | --- |
| Preparation | X | X | X | X |  |  |  |  |  |  |  |  |  |  |  |  |  |  |  |  |  |  |  |  |  |  |  |  |
| Set-up of Assisted CP | X | X | X | X | X | X |  |  |  |  |  |  |  |  |  |  |  |  |  |  |  |  |  |  |  |  |  |  |
| Distribution of curtains |  |  |  | X |  |  |  |  |  |  |  |  |  |  |  |  |  |  |  |  |  |  |  |  |  |  |  |  |
| Entomological evaluation |  |  |  | X | X | X | X | X | X | X | X | X | X | X | X | X | X | X | X | X | X | X | X | X | X | X | X | X |
| Acceptance evaluation |  |  |  |  |  |  | X |  |  |  |  |  |  |  |  |  |  |  |  |  | X |  |  |  |  |  |  |  |
| Uptake evaluation |  |  |  |  | X |  |  |  | X |  |  |  | X |  |  |  | X |  |  |  | X |  |  |  | X |  |  |  |
| Community involvement evaluation | X |  |  |  |  |  |  |  |  |  |  |  |  |  |  |  |  |  |  |  |  | X |  |  |  |  |  |  |
| Adoption of models evaluation |  |  |  |  |  |  |  |  |  |  |  |  |  |  |  |  |  |  |  |  |  | X |  |  |  |  |  |  |
| Economic evaluation household/ individual level |  |  |  |  | X |  | X |  | X |  |  |  | X |  |  |  | X |  |  |  | X |  |  |  | X |  |  |  |
| Economic evaluation programme costing and household collective activities | X | X | X | X | X | X | X | X | X | X | X | X | X | X | X | X | X | X | X | X | X | X | X | X | X | X |  |  |

Reference List

1. WHO HQ, Geneva. Strengthening Implementation of the Global Strategy for Dengue Fever/Dengue Haemorrhagic Fever Prevention and Control. Report of the Informal Consultation 18-20 October 1999. (WHO/CDS/(DEN)/IC/2000.1). 2000.

2. PAHO. Dengue and dengue haemorrhagic fever in the Americas: guidelines for prevention and control. Sci. Pub 548. 1994. Washington DC, Pan American Health Organization.

3. Kroeger A, Lenhart A, Ochoa M, Villegas E, Levy M, Alexander N et al. Effective control of dengue vectors with curtains and water container covers treated with insecticide in Mexico and Venezuela: cluster randomised trials. BMJ 2006;332(7552):1247-52.

4. Focks DA, Brenner RJ, Hayes J, Daniels E. Transmission thresholds for dengue in terms of Aedes aegypti pupae per person with discussion of their utility in source reduction efforts. Am.J.Trop.Med.Hyg. 2000;62(1):11-8.

5. Focks, D. A review of entomological sampling mlethods and indicators for dengue vectors. WHO/TDR/IDE/Den.03.1. 2004.

6. Hayes RJ, Bennett S. Simple sample size calculation for cluster-randomized trials. Int.J.Epidemiol. 1999;28(2):319-26.

***Annex 1: Cluster-allocation***

| **Intervention** | **Circumscriptions** | **Number of blocks/circumscription** | **Number of houses/circumscription** |
| --- | --- | --- | --- |
| **Intervention 1:**  **Routine programme + Curtains** | 45 | 7 | 367 |
|  | 144 | 10 | 356 |
|  | 30 | 9 | 422 |
| **Intervention 2:**  **Routine programme + Curtains + assisted Community participation** | 194 | 12 | 658 |
|  | 124 | 2 | 649 |
|  | 69 | 9 | 433 |
| **Control 1:**  **Routine programme** | 51 | 17 | 791 |
|  | 198 | 4 | 350 |
|  | 48 | 5 | 199 |
| **Control 2:**  **Routine programme+ assisted community participation** | 47 | 12 | 795 |
|  | 38 | 10 | 576 |
|  | 29 | 8 | 410 |
